# Non-*fumigatus Aspergillus*-associated pulmonary events: a diagnostic challenge

**DOI:** 10.1128/jcm.00163-26

**Published:** 2026-03-18

**Authors:** Cendrine Godet, Valentin Joste, Jean-Pierre Frat, Antoine Khalil, Vincent Bunel, Tiphaine Goletto, Thomas Maitre, Armelle Marceau, Domitille Mouren, Lise Morer, Mathilde Salpin, Gaëlle Weisenburger, Camille Taillé, Jacques Cadranel, Hervé Mal, Christine Bonnal

**Affiliations:** 1 Assistance Publique - Hôpitaux de Paris, Hôpital Tenon, Service de Pneumologie et Oncologie Thoracique, Centre constitutif Maladies pulmonaires rares, Sorbonne Université 27063, Paris, France; 2 GRC 40 Sorbonne study group for Lung Infectious Diseases (SoLID), Sorbonne Université 27063, Paris, France; 3 Hôpital Bichat, Laboratoire de Parasitologie Mycologie, Assistance Publique - Hôpitaux de Paris 26930, Paris, France; 4 Équipe Rate, Infections et Parasites, Institut Pasteur 27058, Paris, France; 5 CHU de Poitiers, Médecine Intensive Réanimation, Université de Poitiers, INSERM, CIC-1402, IS-ALIVE 27077, Poitiers, France; 6 Hôpital Bichat, Service de Radiologie, Assistance Publique - Hôpitaux de Paris 26930, Paris, France; 7 Univ. Paris Cité, Assistance Publique - Hôpitaux de Paris, Hôpital Bichat, Service de Pneumologie et Transplantation pulmonaire, Paris, France; University of Utah, Salt Lake City, Utah, USA

**Keywords:** non-*fumigatus Aspergillus*, respiratory samples, *Aspergillus *diagnosis, non-*fumigatus Aspergillus *diseases, serological tests

## Abstract

Diagnosing pulmonary diseases caused by non-*fumigatus Aspergillus* species remains challenging. We conducted a single-center, retrospective observational study in a French Respiratory Medicine Department. Patients with at least one respiratory sample positive for a non-*fumigatus Aspergillus* species, without concurrent *A. fumigatus* isolation over a 12-month period, were included. The primary objective was to determine the prevalence of pulmonary events (colonization or pulmonary diseases) associated with non-*fumigatus Aspergillus* species. Secondary objectives included species characterization and assessment of positive results for available diagnostic tests, including direct examination and fungal culture from respiratory samples, galactomannan in bronchoalveolar lavage, and serum *A. fumigatus*-specific IgG. Between April 2017 and January 2022, 497 patients (39.6%) had cultures positive for non-*fumigatus Aspergillus* species. Among them, 52 (10.5%) experienced pulmonary events: 36 were colonized, and 16 had a documented pulmonary disease. *Aspergillus niger* was the most frequently isolated species (41%), followed by *Aspergillus flavus* (27%) and *Aspergillus nidulans* (10%). Positive results were observed in 10/437 (2.3%) samples for direct examination, 75/808 (9.3%) for fungal culture, 7/94 (7.4%) for galactomannan in bronchoalveolar lavage, and 12/49 (24.5%) for serum *Aspergillus fumigatus*-specific IgG. Among patients with *non-fumigatus Aspergillus* positive respiratory samples, most were colonized, while nearly one-third had clinically significant pulmonary diseases, underscoring the clinical relevance of these species. Low positivity rates across diagnostic tests underscore the need for repeated respiratory sampling and fungal culture and suggest that assays primarily designed for *A. fumigatus* may under-detect these pulmonary events.

**IMPORTANCE:**

Non-*fumigatus Aspergillus* species are recognized as pulmonary pathogens, but their diagnosis is poorly documented. In this 5-year, single-center study, 497 of 1,256 patients had at least one positive respiratory culture for a non-*fumigatus* species, with 36 considered colonized and 16 with documented lung disease. *A. niger*, *A. flavus,* and *A. nidulans* accounted for almost four-fifths of the isolates. Routine tests produced poor results, with positivity rates of 2.3% for microscopy, 9.3% for repeat culture, 7.4% for bronchoalveolar galactomannan, and 24.5% for *Aspergillus fumigatus* serum IgG. Overall, the study shows that non-*fumigatus* species can cause treatable chronic lung disease, but that current diagnostics miss most cases. Until more sensitive tests are available, clinicians must rely on repeated respiratory sampling and culture to identify these infections.

## INTRODUCTION

*Aspergillus* is a ubiquitous genus of filamentous fungi capable of causing a wide spectrum of human diseases, particularly in individuals with comorbidities, immunosuppressive therapy, or structural lung abnormalities. In these conditions, colonization can progress to sensitization or overt respiratory infection. Depending on the host immune response, *Aspergillus* species may cause pulmonary events ranging from asymptomatic colonization to clinically significant diseases, including acute and subacute invasive pulmonary aspergillosis (IPA), chronic pulmonary aspergillosis (CPA), and allergic bronchopulmonary aspergillosis (ABPA) (1).

The primary *Aspergillus* species involved in pulmonary aspergillosis belong to the *Fumigati*, *Flavi*, *Nigri*, and *Terrei* sections (2). Differences in pathogenicity among *Aspergillus* species are influenced by conidial size, virulence, germination rate, adherence, and resistance to phagocytosis (3). *A. fumigatus* is the predominant agent of IPA, followed by *A. flavus* and *A. terreus* (4, 5). Non-*fumigatus* species, such as *A. niger* and *A. flavus*, have also been reported in CPA and ABPA although their epidemiology remains poorly characterized and likely underreported (2, 6). Species-level identification is critical for guiding antifungal therapy, as resistance profiles vary widely, and the increasing prevalence of triazole resistance underscores the need for targeted susceptibility testing (7). Early screening, diagnosis, and appropriate treatment are crucial to reducing the global burden of *Aspergillus*-related pulmonary diseases (8).

Diagnosis relies primarily on respiratory sample culture and direct microscopic examination, which allow species-level identification, complemented by serological assays such as *A. fumigatus*-specific IgG. While molecular assays and antigen detection methods are widely used for *A. fumigatus*, their diagnostic performance for non-*fumigatus* species remains limited, highlighting a critical knowledge gap (8–11).

The European Organization for Research and Treatment of Cancer/Mycoses Study Group (EORTC/MSG) criteria provide a standardized framework for classifying pulmonary aspergillosis cases as proven, probable, or possible based on host factors, clinical features, and mycological evidence (12). However, these criteria have limitations for non-immunocompromised patients and non-*fumigatus* species, reflecting broader uncertainties in diagnosis. Similarly, European guidelines for CPA and ABPA offer limited specificity for non-*fumigatus* infections (10, 13).

To address these limitations, the present study aims to characterize the period prevalence of pulmonary events associated with non-*fumigatus Aspergillus* species. Secondary objectives include detailed species characterization and assessment of positive results for available diagnostic tests.

## MATERIALS AND METHODS

### Study design

We conducted a single-center, retrospective observational study in the Department of Respiratory Medicine at Bichat University Hospital (AP-HP, Paris, France). The study was approved by the Institutional Review Board of the Société de Pneumologie de Langue Française and by the Comité d’Évaluation des Protocoles de Recherche Observationnelle (CEPRO 2022-049).

### Study participants

Between April 2017 and January 2022, patients with respiratory cultures positive for *Aspergillus* species were screened. Eligible patients had at least one respiratory sample positive for a non-*fumigatus Aspergillus* species from sputum, bronchial aspirate (BA), or bronchoalveolar lavage (BAL).

Patients were included if, within 6 months before or after the initial positive non-*fumigatus* culture, they met one of the following criteria:

1. At least two bronchoscopy samples;
2. One bronchoscopy and one sputum sample;
3. Three sputum samples.

Patients with any isolation of *A. fumigatus* during the 12-month observation period were excluded. The 6-month observation window, before and after the index isolation, was defined to capture clinically significant non-*fumigatus* isolates while excluding contamination or co-infection. Samples obtained within 3 months before or after the diagnosis of a non-*fumigatus* pulmonary event were considered attributable to that event.

### Immunosuppression

Immunosuppression was defined according to EORTC/MSG criteria as the presence of one or more of the following: systemic corticosteroid therapy equivalent to ≥0.3 mg/kg/day prednisone for ≥3 weeks within 60 days prior to sampling; cytotoxic chemotherapy; calcineurin inhibitors; anti-TNF agents or other immunomodulators; hematologic malignancy; or solid organ/stem cell transplantation (12).

### Classification of pulmonary events and clinical definition

Pulmonary events associated with non-*fumigatus Aspergillus*-positive cultures were classified as colonization or disease. Each event was defined by composite clinical, radiological, and laboratory criteria, and only samples collected within 3 months before or after the pulmonary diagnosis were attributed to that event.

Patients were classified as follows, encompassing the spectrum of pulmonary manifestations associated with non-*fumigatus Aspergillus* (9, 10, 13, 14):

- **Colonization:** Isolation of non-*fumigatus Aspergillus* in respiratory samples without radiological progression or serological markers over ≥3 months of follow-up.
- ***Aspergillus* Bronchitis:** Persistent cough and sputum production with repeated isolation of non-*fumigatus Aspergillus*, without radiological evidence of parenchymal invasion or ABPA criteria.
- **CPA**: Chronic symptoms (>3 months), radiological findings compatible with CPA (cavities, nodules, and pleural thickening), and positive mycological evidence of non-*fumigatus Aspergillus*.
- **ABPA:** Asthma, elevated total IgE, positive specific IgE or skin tests for non-*fumigatus Aspergillus*, and radiological features (infiltrates, central bronchiectasis).
- **Overlap Syndromes** (CPA + ABPA): Patients meeting criteria for both CPA and ABPA concurrently.
- **IPA**: Immunocompromised patients or those with significant pulmonary risk factors, compatible symptoms, radiological evidence, and microbiological confirmation of non-*fumigatus Aspergillus*, including invasive tracheobronchial forms with histopathologic or microbiologic evidence of mucosal or submucosal airway invasion.

### Microbiological and diagnostic laboratory tests for aspergillosis

Direct microscopic examination and fungal culture were performed on respiratory samples including sputum, BA, and BAL. Galactomannan antigen testing was conducted exclusively on BAL samples. Direct examination was not systematically performed for all specimens due to sample volume limitations and prioritization for culture.

#### *Aspergillus* species identification: direct examination and fungal culture

Direct examination was carried out using a 10% potassium hydroxide solution (Uvibio, LDBio Diagnostics, Lyon, France), following standard recommendations (9). A sample was considered positive when septate hyphae suggestive of *Aspergillus* spp. were visualized (4). All respiratory specimens were cultured on Sabouraud chloramphenicol gentamicin agar (Bio-Rad, Marne-la-Coquette, France) and incubated at 35°C and 27°C for 7 days (sputum and BA) and 14 days (BAL).

Filamentous fungi were identified by macro- and microscopic evaluation, using the flagging technique and staining with Lactophenol blue (Merck KGaA, Darmstadt, Germany) to visualize fungal structures. Species-level identification was confirmed using matrix-assisted laser desorption/ionization time-of-flight mass spectrometry (MALDI-TOF MS; Bruker MALDI Biotyper, Bruker France, Champ-sur-Marne, France) and the MSI database (version 2.0)**,** which includes cryptic *Aspergillus* species (15).

#### *Aspergillus* antigen

Galactomannan (GM) antigen was detected in BAL using the Sona *Aspergillus* GM Lateral Flow Assay (IMMY, Norman, OK, USA), following the manufacturer’s instructions. Briefly, 300 µL of BAL fluid was mixed with 100 µL of pre-treatment solution and heated at 120°C for 7 min. After centrifugation, 80 µL of supernatant was combined with 40 µL of migration buffer and applied to the test strip. After 30 min of incubation, results were read quantitatively with the Sona Reader Cube. A threshold index of ≥1 was considered positive consistent with previous validation studies for BAL GM testing (16).

#### *Aspergillus* IgG antibody

We employed an enzyme-linked immunosorbent assay (ELISA) (PLATELIA *Aspergillus* IgG, Bio-Rad, Marne-la-Coquette, France). A positive result for *A. fumigatus*-specific IgG was defined as a concentration ≥10 UA/mL in accordance with the manufacturer’s recommendations and prior studies on CPA diagnosis (17).

Although the primary assay is designed to detect *A. fumigatus*-specific antibodies, it has been shown to identify cross-reactive antibodies in infections caused by other *Aspergillus* species due to shared antigenic epitopes (18, 19).

### Statistical analysis

Qualitative data are expressed as numbers and percentages. Quantitative variables are presented as means ± standard deviation (SD). Continuous variables were compared using the unpaired Student’s *t*-test or the Mann–Whitney *U* test when the assumption of normality was not met. Categorical variables were compared using the chi-square test or Fisher’s exact test when expected counts were small. A *P*-value < 0.05 was considered statistically significant. All statistical analyses were performed using SAS software (version 9.4; SAS Institute, Cary, NC, USA).

## RESULTS

### Patient characteristics

Between April 2017 and January 2022, respiratory cultures were positive for *Aspergillus* species in 1,256 patients: 642 out of 1,256 (51.1%) had culture positive for *Aspergillus fumigatus*, 497/1,256 (39.6%) for non-*fumigatus Aspergillus* species, and 117/1,256 (9.3%) for both.

**Fig 1:**
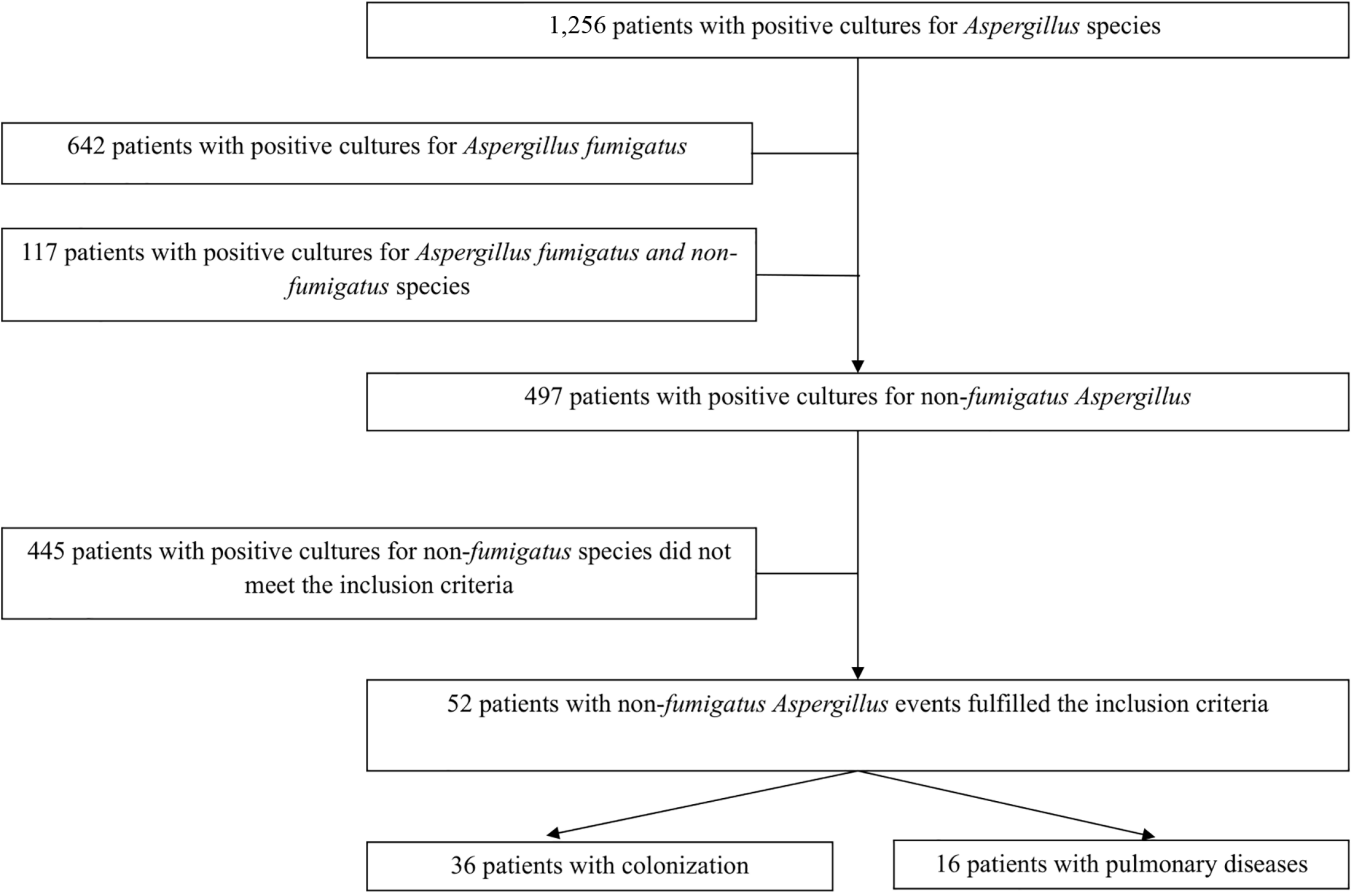
Flowchart of patient enrollment.

Among the 497 patients with at least one culture positive for non-*fumigatus Aspergillus* species, 52/497 (10.5%) patients met the inclusion criteria and were identified as having undergone non-*fumigatus Aspergillus*-associated pulmonary events: 36 were classified as colonized, and 16 had a documented pulmonary disease (4 *Aspergillus* bronchitis, 4 CPA, 2 *Aspergillus* nodules, 3 ABPA, 2 overlap syndromes, and 1 IPA) (Fig. 1). A supplementary Table summarizes the baseline characteristics of included patients (Table S1).

Immunosuppressive therapy was reported in 42 out of 52 patients (80.8%), including high-dose corticosteroids (≥0.5 mg/kg/day for ≥3 weeks) or other immunosuppressive agents such as calcineurin inhibitors or mycophenolate mofetil. The most common underlying condition was lung transplantation in 39/52 patients (75%), including 16 single-lung (30.8%) and 23 bilateral-lung (44.2%) transplant recipients. Chronic kidney disease or diabetes mellitus was present in 22/52 patients (42.3%) (Table 1).

**TABLE 1:** Clinical characteristics of patients with pulmonary events associated with non-*fumigatus Aspergillus* species

Characteristics	All patients (*n* = 52)	Patients with colonization (*n* = 36)	Patients with pulmonary diseases (*n* = 16)
Male sex, *n* (%)	37 (71.2)	28 (77.8)	9 (56.3)
Age (years), median (IQR)	60 (13.9)	59 (13.8)	60 (12.9)
Underlying lung disease, *n* (%)			
Single-lung transplant recipient	16 (30.8)	12 (33.3)	4 (25)
Bilateral-lung transplant recipient	23 (44.2)	20 (55.5)	3 (18.8)
Chronic obstructive pulmonary disease	4 (7.7)	2 (5.5)	2 (12.5)
Interstitial lung disease	1 (1.9)	1 (2.8)	0 (0)
Asthma	2 (3.8)	0 (0)	2 (12.5)
Prior pulmonary tuberculosis sequelae	2 (3.8)	0 (0)	2 (12.5)
Others	4 (7.7)	1 (2.8)	3 (18.8)
Comorbidities, *n* (%)			
Diabetes mellitus	22 (42.3)	19 (52.8)	3 (18.8)
Chronic renal failure	22 (42.3)	19 (52.8)	3 (18.8)
Neutropenia	6 (11.5)	5 (13.9)	1 (6.3)
Immunosuppressive treatments, *n* (%)			
Systemic corticosteroids	42 (80.8)	34 (94.4)	8 (50)
Inhaled corticosteroids	7 (13.5)	2 (5.5)	5 (31.3)
Immunosuppressive agents	40 (76.9)	33 (91.7)	7 (43.8)
Previous antifungal treatment, *n* (%)	15 (28.8)	12 (33.3)	3 (18.8)
Azole	12 (23.1)	10 (27.8)	2 (12.5)
Inhaled liposomal amphotericin B	3 (5.8)	2 (5.5)	1 (6.3)
Active antifungal treatment, *n* (%)	28 (53.8)	22 (61.1)	6 (37.5)
Azole	19 (36.5)	15 (41.7)	4 (25)
Inhaled liposomal amphotericin B	9 (17.3)	7 (19.4)	2 (12.5)

### Species distribution

**Fig 2:**
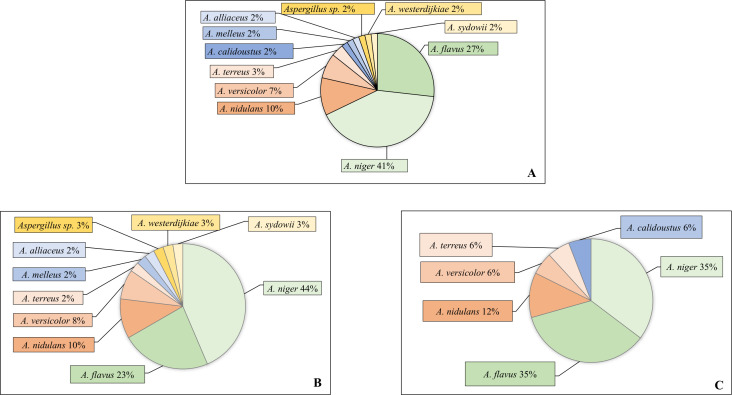
Distribution of the non-*fumigatus Aspergillus* species among patients with pulmonary events (colonization and pulmonary diseases): (**A**) 56 non-*fumigatus Aspergillus* strains isolated from 52 patients with non-*fumigatus Aspergillus*-associated pulmonary events; (**B**) 39 non-*fumigatus Aspergillus* strains isolated from 36 colonized patients; (**C**) 17 non-*fumigatus Aspergillus* strains isolated from 16 patients with pulmonary diseases.

Among 52 patients with pulmonary events associated with non-*fumigatus Aspergillus* species, 56 non-*fumigatus Aspergillus* isolates were identified. The most frequently isolated species were *A. niger* (23/56, 41%), *A. flavus* (15/56, 27%), and *A. nidulans* (6/56, 10%) (Fig. 2).

### Direct examination and culture

During the 12-month study period, a total of 808 respiratory samples were collected from 52 patients with non-*fumigatus Aspergillus*-associated pulmonary events, including 98 sputum samples, 190 BAL specimens, and 520 BA.

Direct microscopic examination was positive in 10/437 samples (2.3%), whereas fungal culture yielded positive results in 75/808 samples (9.3%) (Tables 2 and 3). Sputum samples showed higher culture positivity rates (16/98, 16.3%) compared with bronchial aspirates (44/520, 8.5%) and BAL specimens (15/190, 7.9%) (Table 2). Overall, fungal cultures were significantly more often positive in case of non-*fumigatus Aspergillus*-associated pulmonary events than in colonized patients (18.0% vs 6.7%; *P* < 0.001).

**TABLE 2:** Identification of *Aspergillus* species and corresponding positive laboratory findings in the 52 non-*fumigatus Aspergillus*-associated pulmonary events

*Aspergillus* species identification*,* number of patients^*a*^	Direct examination	Sputum culture	Bronchial aspiration culture	BAL culture	BAL-GM	Serum total IgG
*A. niger, n* = 22	0/183	6/42	16/244	4/64	2/28	7/18
*A. flavus, n* = 11	9/87	7/26	10/82	7/44	5/22	3/11
*A. nidulans*, *n* = 4	0/16	1/9	3/15	2/3	0/2	0/3
*A. versicolor*, *n* = 4	0/35	0/2	3/31	1/31	0/16	0/1
*A. terreus*, *n* = 2	0/28	1/3	2/32	0/13	0/7	0/2
*A. alliaceus*, *n* = 1	0/25	0/0	1/32	0/14	0/7	0/1
*A. calidoustus*, *n* = 1	0/5	1/6	0/2	0/2	0/1	0/1
*A. melleus, n* = 1	0/9	0/1	2/19	0/0	0/0	0/2
*A*. sp.*, n* = 1	0/5	0/6	1/2	0/0	0/0	0/1
*A. westerdijkiae, n* = 1	1/12	0/0	1/18	0/5	0/3	0/2
*A. nidulans* and *A. flavus*, *n* = 2	0/21	0/0	2/32	0/10	0/5	2/2
*A. niger* and *A. flavus, n* = 1	0/7	0/3	1/5	1/3	0/2	0/3
*A. sydowii* and *A. flavus*, *n* = 1	0/4	0/0	2/6^*b*^	0/1	0/1	0/2
Positive cultures or tests, *n*/*n* total (%)	10/437 (2.3)	16/98 (16.3)	44/520 (8.5)	15/190 (7.9)	7/94 (7.4)	12/49 (24.5)

^*a*^ Patients are grouped according to the *Aspergillus* species isolated. For each group, positive results are shown as a ratio of the number of tests performed.

^*b*^ One bronchial aspirate was positive with both *Aspergillus* species, and another for *A. flavus* only. BAL: Bronchoalveolar lavage; GM: galactomannan antigen.

**TABLE 3:** Positive laboratory test results in the 16 patients with non-*fumigatus Aspergillus* pulmonary disease and the 36 with colonization^*a*^^,^^*b*^^,^^*c*^

Laboratory tests	IPA (*n* = 1)	CPA (*n* = 4)	A. nodule (*n* = 2)	ABPA (*n* = 3)	A. bronchitis (*n* = 4)	Overlaps syndrome (*n* = 2)	Colonized patients (*n* = 36)	Total (*n* = 52)
Direct examination, *n*/*n* total								
Samples Patients	9/22 1/1 (100)	0/24 (0) 0/4 (0)	0/9 (0) 0/2 (0)	0/11 (0) 0/3 (0)	0/33 (0) 0/4 (0)	0/11 (0) 0/2 (0)	1/327 (0) 1/36 (3)	10/437 (2) 2/52 (4)
Culture								
Samples Patients	10/38 (26) 1/1 (100)	6/42 (14) 4/4 (100)	3/14 (21) 2/2 (100)	4/21 (19) 3/3 (100)	5/52 (10) 4/4 (100)	5/16 (31) 2/2 (100)	42/625 (7) 36/36 (100)	75/808 (9) 52/52 (100)
Species involved	*A. flavus*	*A. flavus* *A. versicolor* *A. nidulans*	*A. flavus* *A. terreus*	*A. nidulans* *A. calidoustus* *A. niger*	*A. flavus* *A. niger*	*A. niger*	*A*. spp^*d*^	
BAL-galactomannan								
Samples Patients	5/10 (50) 1/1 (100)	0/6 (0) 0/2 (0)	0/2 (0) 0/2 (0)	0/2 (0) 0/2 (0)	0/3 (0) 0/2 (0)	0/1 (0) 0/1 (0)	2/70 (2.9) 2/18 (11.1)	7/94 (7.4) 3/28 (10.7)
*A. fumigatus* IgG antibody								
Samples Patients	0/1 (0) 0/1 (0)	0/3 (0) 0/2 (0)	0/1 (0) 0/1 (0)	1/3 (33) 1/3 (33)	2/6 (33) 1/3 (33)	1/1 (100) 1/1 (100)	8/34 (23) 4/23 (17)	12/49 (24) 7/34 (21)

^*a*^ IPA, invasive pulmonary aspergillosis; CPA, chronic cavitary pulmonary aspergillosis; A.nodule, aspergillosis nodule; ABPA: Allergic Bronchopulmonary Aspergillosis; A. bronchitis, aspergillosis bronchitis.

^*b*^ BAL, bronchoalveolar lavage; *A. fumigatus*, *Aspergillus fumigatus.*

^*c*^ Laboratory tests are presented as positive tests/total performed (%).

^*d*^ *A. flavus, A. versicolor, A. nidulans, A. niger, A. terreus, A. melleus, A. alliaceus, A. sydowii, A. westerdijkiae, Aspergillus sp*.

Among the 52 patients with non-*fumigatus Aspergillus*-associated pulmonary events, at least 1 sputum sample was collected from 27 patients, of whom 14 (51.9%) had at least one positive culture. Bronchial aspirates were obtained from 49 patients, with 36 patients (73.5%) showing at least 1 positive culture. BAL was performed in 34 patients, among whom 12 (35.3%) had a positive culture.

### Serology

Serum *Aspergillus fumigatus*-specific IgG was detected in 12 out of 49 samples (24.5%), corresponding to 7 among 34 patients (20.6%) who underwent serological testing (Table 3).

### Galactomannan in BAL

Galactomannan testing in BAL specimens showed a low rate of positivity, with 7/94 samples (7.4%) testing positive, corresponding to 3 among 28 patients (10.7%) in whom the assay was performed.

## DISCUSSION

In this single-center study, non-*fumigatus Aspergillus* species accounted for nearly 40% of isolates and were identified as potential etiologic agents of pulmonary diseases. Most patients were lung transplant recipients or individuals with pre-existing pulmonary conditions, reflecting the high-risk population of our center. Both groups are known to be at increased risk for invasive or chronic pulmonary aspergillosis, depending on the degree of immunosuppression (20).

Patients with non-*fumigatus* pulmonary infections frequently received systemic corticosteroids or other immunosuppressive therapies and had comorbidities such as diabetes mellitus or chronic kidney disease, consistent with known risk factors for pulmonary aspergillosis (10). Lung transplant recipients were more commonly colonized than patients with other pulmonary diseases, likely reflecting the routine use of antifungal prophylaxis, which mitigates invasive infection but allows persistent colonization (21, 22).

The most frequently isolated non-*fumigatus* species were *A. niger* and *A. flavus*. This finding is consistent with a recent study by Takeda et al., which reported that the *A. niger* species complex and *A. flavus* accounted for 18% and 3% of pulmonary aspergillosis cases, respectively, compared with 75% for *A. fumigatus* (2). Stemler et al. reported an increasing incidence of invasive aspergillosis due to the *A. niger* complex, with rates ranging from 0.23 to 0.95 per 100,000 patient-days between 2005 and 2011 (4). *A. flavus* is the second most frequently isolated *Aspergillus* species in invasive aspergillosis cases reported from warm and arid regions, particularly in subtropical countries such as India, Pakistan, Saudi Arabia, Tunisia, and Mexico most likely owing to its thermotolerance (4, 23). The catchment area of our hospital includes a substantial number of patients from these regions, which may partly explain the higher isolation rates of this species. *A. flavus* exhibits higher minimum inhibitory concentrations (MICs) to amphotericin B (24). Increasing use of nebulized amphotericin B in recent years, particularly for prophylaxis in lung transplant recipients and treatment of chronic aspergillosis, may favor the selection and persistence of *A. flavus* in these patients. *A. nidulans* is most frequently associated with primary immunodeficiencies, notably chronic granulomatous disease, which are characterized by NADPH oxidase deficiency (25). However, in our study, *A. nidulans* was the third most frequently isolated species despite a range of immunosuppressive conditions, suggesting that factors beyond NADPH oxidase deficiency may predispose patients to pulmonary infection with *A. nidulans*. While *A. fumigatus* is the predominant cause of *Aspergillus* bronchitis, other species such as *A. niger*, *A. terreus*, and *A. flavus* have also been implicated (26).

Direct examination and culture of respiratory specimens were infrequently positive, with sputum yielding higher proportion of positive results than BA and BAL. These findings mirror previous reports indicating culture and microscopy positivity rates below 30% and 10%, respectively, for non-*fumigatus* infections (2). Similarly, culture positivity in patients with CPA ranges from 17% to 32% (26). Despite the low yield of fungal cultures, they remain essential for species identification, detection of azole resistance, and assessment of treatment response (15, 27). Repeated, high-quality respiratory sampling is, therefore, recommended, including undiluted or high-volume sputum cultures whenever feasible (26).

*A. fumigatus*-specific IgG was positive in a subset of patients with non-*fumigatus Aspergillus* infections. This likely reflects cross-reactivity between *Aspergillus* species rather than true co-infection, as most IgG assays are primarily validated for *A. fumigatus*. Such cross-reactivity highlights the challenge of interpreting serologic results in the context of non-*fumigatus* infections and emphasizes the need to interpret serological results cautiously and in conjunction with culture and clinical findings. Moreover, negative serology should not exclude infection, and repeated sampling combined with culture and clinical assessment remains essential.

Galactomannan testing in BAL specimens showed low positivity in our cohort, highlighting the limited sensitivity of this assay for detecting non-*fumigatus Aspergillus* pulmonary events. Negative results should, therefore, be interpreted with caution, and diagnosis should rely on repeated respiratory sampling, culture, and clinical assessment.

Accurate species identification is essential for guiding antifungal therapy. Although antifungal susceptibility testing was not performed in this study, previous reports indicate that some *A. flavus* isolates exhibit amphotericin B MICs >2 mg/L, and pan-azole resistance has been observed in 2.5%–5% of isolates (28). For *A. niger*, azole monotherapy may be suboptimal due to elevated MICs, further emphasizing the importance of precise species-level identification.

The strengths of this study include the careful definition by composite clinical, radiological, and laboratory criteria and selection of pulmonary events clearly associated with non-*fumigatus Aspergillus* species, minimizing the risk of co-infection or colonization. In addition, the hospital’s catchment area exposes patients to a broad range of non-fumigatus *Aspergillus* species, and repeated sampling increases the likelihood of detecting clinically relevant events. This study has several limitations. It is a single-center, retrospective analysis reflecting local clinical practices among lung transplant recipients. The higher number of endobronchial samples and the profound immunosuppression observed in transplant recipients may limit the generalizability of our findings to non-transplant populations. The small sample size also precluded interspecies comparisons.

In conclusion, pulmonary diseases requiring clinical management may involve non-*fumigatus Aspergillus* species. Given the limited diagnostic yield of current microbiological and serological assays, clinicians should not rely solely on these tests. Repeated respiratory sampling, culture-based identification, and careful clinical assessment remain the cornerstone of diagnosis, with awareness of potential serologic cross-reactivity.

## SUPPLEMENTAL MATERIAL

Table S1 jcm.00163-26-s0001.docx Clinical and biological characteristics of the 52 patients.

ASM does not own the copyrights to Supplemental Material that may be linked to, or accessed through, an article. The authors have granted ASM a non-exclusive, world-wide license to publish the Supplemental Material files. Please contact the corresponding author directly for reuse.
