# Supplementary material for: Non-fumigatus Aspergillus-associated pulmonary events: a diagnostic challenge
Source: J Clin Microbiol. 2026 Mar 18;64(4):e00163-26. doi: 10.1128/jcm.00163-26 (PMC13059719; doi:10.1128/jcm.00163-26)
Supplement: Table S1 — Clinical and biological characteristics of the 52 patients. [file jcm.00163-26-s0001.docx]

Table S1: Clinical and biological characteristics of the 52 patients

| Patients | Underlying conditions | Inclusion period | *Aspergillus* species | Positive DE/total DE | Positive cultures/  total cultures | Positive GM in BAL/  total BAL | Positive IgG/  total IgG | Diagnosis |
| --- | --- | --- | --- | --- | --- | --- | --- | --- |
| 1 | bilateral lung transplant | 28/02/2020-29/08/2020 | *Aspergillus sp* | 0/5 | 1/8 | 0/0 | 0/1 | Colonization |
| 2 | bilateral lung transplant | 21/02/2021-21/08/2021 | *A niger* | 0/17 | 1/31 | 0/7 | 0/2 | Colonization |
| 3 | chronic obstructive pulmonary disease | 12/05/2021-12/11/2021 | *A flavus* | 0/15 | 1/23 | 0/4 | 3/3 | Colonization |
| 4 | single lung transplant | 28/04/2020-28/10/2020 | *A versicolor* | 0/24 | 1/46 | 0/14 | 0/1 | Colonization |
| 5 | single lung transplant | 06/10/2021-06/04/2022 | *A flavus,*  *A sydowii* | 0/4 | 2/7 | 0/1 | 0/2 | Colonization |
| 6 | single lung transplant | 25/01/2019-25/07/2019 | *A niger* | 0/12 | 1/25 | 0/3 | 0/0 | Colonization |
| 7 | single lung transplant | 28/07/2020-28/01/2021 | *A versicolor* | 0/6 | 1/10 | 0/0 | 0/0 | Colonization |
| 8 | single lung transplant | 29/05/2019-29/11/2019 | *A niger* | 0/16 | 1/27 | 1/2 | 0/1 | Colonization |
| 9 | single lung transplant | 04/06/2020-04/12/2020 | *A nidulans* | 0/4 | 1/7 | 0/1 | 0/1 | Colonization |
| 10 | bilateral lung transplant | 15/11/2020-15/05/2021 | *A niger* | 0/19 | 1/40 | 0/2 | 1/1 | Colonization |
| 11 | bilateral lung transplant | 01/09/2020-01/03/2021 | *A niger* | 0/18 | 2/36 | 0/8 | 2/2 | Colonization |
| 12 | asthma | 01/09/2020-01/03/2021 | *A niger* | 0/3 | 1/5 | 0/0 | 1/1 | Allergic bronchopulmonary aspergillosis |
| 13 | asthma | 23/09/2020-23/03/2021 | *A nidulans* | 0/3 | 2/6 | 0/1 | 0/1 | Allergic bronchopulmonary aspergillosis |
| 14 | single lung transplant | 10/08/2021-10/02/2022 | *A niger* | 0/5 | 1/9 | 0/0 | 0/1 | Colonization |
| 15 | bilateral lung transplant | 26/10/2020-26/04/2021 | *A niger* | 0/3 | 1/5 | 0/1 | 0/0 | Colonization |
| 16 | bilateral lung transplant | 25/03/2021-25/09/2021 | *A versicolor* | 0/1 | 1/2 | 0/0 | 0/0 | Colonization |
| 17 | bilateral lung transplant | 25/06/2021-25/12/2021 | *A flavus* | 0/3 | 2/4 | 0/0 | 0/1 | Colonization |
| 18 | single lung transplant | 18/11/2020-18/05/2021 | *A westerdijkiae* | 1/12 | 1/23 | 1/3 | 0/2 | Colonization |
| 19 | bilateral lung transplant | 16/09/2021-16/03/2022 | *A flavus* | 9/22 | 10/38 | 5/10 | 0/1 | Invasive pulmonary aspergillosis |
| 20 | bilateral lung transplant | 29/06/2021-29/12/2021 | *A alliaceus* | 0/25 | 1/46 | 0/7 | 0/1 | Colonization |
| 21 | mucoviscidosis | 27/03/2021-27/09/2021 | *A calidoustus* | 0/5 | 1/10 | 0/1 | 0/1 | Allergic bronchopulmonary aspergillosis |
| 22 | single lung transplant | 09/06/2020-09/12/2020 | *A niger* | 0/4 | 1/8 | 0/0 | 0/0 | Colonization |
| 23 | bilateral lung transplant | 15/07/2021-15/01/2022 | *A niger* | 0/9 | 1/21 | 0/0 | 0/0 | Colonization |
| 24 | bilateral lung transplant | 06/02/2020-06/08/2020 | *A nidulans, A flavus* | 0/19 | 1/38 | 0/5 | 2/2 | Colonization |
| 25 | bilateral lung transplant | 06/06/2021-06/12/2021 | *A terreus* | 0/23 | 1/40 | 0/6 | 0/2 | Colonization |
| 26 | single lung transplant | 09/12/2019-09/06/2020 | *A flavus* | 0/13 | 2/25 | 0/4 | 0/0 | Chronic cavitary pulmonary aspergillosis |
| 27 | bilateral lung transplant | 05/08/2020-05/02/2021 | *A niger* | 0/2 | 1/4 | 0/0 | 0/2 | Colonization |
| 28 | single lung transplant | 11/07/2019-11/01/2020 | *A nidulans* | 0/4 | 2/7 | 0/0 | 0/0 | Colonization |
| 29 | chronic obstructive pulmonary disease | 13/09/2019-13/03/2020 | *A flavus* | 0/2 | 1/6 | 0/1 | 0/1 | Colonization |
| 30 | chronic obstructive pulmonary disease | 30/07/2018-30/01/2019 | *A flavus* | 0/2 | 2/4 | 0/0 | 0/2 | Chronic cavitary pulmonary aspergillosis |
| 31 | single lung transplant | 08/05/2019-08/11/2019 | *A niger* | 0/12 | 1/21 | 0/1 | 0/1 | *Aspergillus* bronchitis |
| 32 | single lung transplant | 12/02/2019-12/08/2019 | *A flavus* | 0/4 | 1/5 | 0/0 | 0/0 | *Aspergillus* bronchitis |
| 33 | bronchial dilatation | 26/08/2019-26/02/2020 | *A flavus* | 0/4 | 1/7 | 0/0 | 0/1 | Colonization |
| 34 | emphysema | 16/09/2018-16/03/2019 | *A terreus* | 0/5 | 2/8 | 0/1 | 0/0 | *Aspergillus* nodules |
| 35 | chronic obstructive pulmonary disease | 04/05/2019-04/11/2019 | *A niger* | 0/10 | 1/15 | 0/0 | 2/2 | *Aspergillus* bronchitis |
| 36 | single lung transplant | 11/11/2018-11/05/2019 | *A niger* | 0/4 | 1/8 | 0/0 | 0/0 | Colonization |
| 37 | single lung transplant | 20/05/2019-20/11/2019 | *A niger* | 0/3 | 1/6 | 0/0 | 0/2 | Colonization |
| 38 | single lung transplant | 08/04/2020-08/10/2020 | *A versicolor* | 0/4 | 1/6 | 0/2 | 0/0 | Chronic cavitary pulmonary aspergillosis |
| 39 | tuberculosis | 26/04/2019-26/10/2019 | *A nidulans* | 0/5 | 1/7 | 0/0 | 0/1 | Chronic cavitary pulmonary aspergillosis |
| 40 | bilateral lung transplant | 27/05/2019-27/11/2019 | *A niger* | 0/4 | 1/8 | 0/0 | 0/0 | Colonization |
| 41 | bilateral lung transplant | 12/09/2019-12/03/2020 | *A melleus* | 0/9 | 2/20 | 0/0 | 0/2 | Colonization |
| 42 | diffuse interstitial lung disease | 06/06/2019-06/12/2019 | *A flavus* | 0/16 | 1/30 | 0/2 | 0/1 | Colonization |
| 43 | bilateral lung transplant | 21/11/2018-21/05/2019 | *A niger* | 0/7 | 1/14 | 0/0 | 0/0 | Colonization |
| 44 | bilateral lung transplant | 06/01/2017-06/07/2017 | *A niger* | 0/9 | 1/20 | 0/1 | 0/1 | Colonization |
| 45 | bilateral lung transplant | 08/05/2018-08/11/2018 | *A flavus* | 0/2 | 2/4 | 0/0 | 0/0 | Colonization |
| 46 | bronchial dilatation | 20/08/2019-20/02/2020 | *A niger* | 0/2 | 2/3 | 0/0 | 1/1 | Overlaps syndrome |
| 47 | bilateral lung transplant | 09/09/2019-09/03/2020 | *A niger* | 0/8 | 1/16 | 1/2 | 0/1 | Colonization |
| 48 | bilateral lung transplant | 02/10/2017-02/04/2018 | *A niger* | 0/7 | 1/15 | 0/0 | 0/0 | Colonization |
| 49 | bilateral lung transplant | 29/05/2019-29/11/2019 | *A flavus* | 0/4 | 1/6 | 0/1 | 0/1 | *Aspergillus* nodules |
| 50 | bilateral lung transplant | 21/10/2018-21/04/2019 | *A nidulans, A flavus* | 0/2 | 1/4 | 0/0 | 0/0 | Colonization |
| 51 | tuberculosis | 13/11/2018-13/05/2019 | *A niger* | 0/9 | 3/13 | 0/1 | 0/0 | Overlaps syndrome |
| 52 | bilateral lung transplant | 19/09/2019-19/03/2020 | *A niger,*  *A flavus* | 0/7 | 2/11 | 0/2 | 0/3 | *Aspergillus* bronchitis |
